# Elements of Medical Statistics

**Published:** 1829-10-01

**Authors:** 


**ELEMENTS OF MEDICAL STATISTICS; CONTAINING THE SUBSTANCE OF THE GULSTONIAN LECTURES, &c. WITH NUMEROUS ADDITIONS ILLUSTRATING THE COMPARATIVE SALUBRITY, LONGEVITY, MORTALITY, AND PREVALENCE OF DISEASES IN THE PRINCIPAL COUNTRIES AND CITIES OF THE CIVILIZED WORLD. By *F. Bisset Hawkins,* M.D. &c. &c. 8vo. pp. 234. 1829.**

IT is a melancholy, but certainly a useful labour to enquire into the causes which shorten man's brief span of existence in this sublunary scene. There can be no doubt that Nature designed that the whole of the three-score years and ten should be enjoyed by the human species, and that all men should ultimately die the DEATH of NATURE. Yet, little more than one half of those who see the light of Heaven are found to run even this short race.

"Independently of the light which this study throws upon Medical Science, it affords the most valuable illustrations of the history, manners, and customs of mankind, and a just criterion of the progressive or retrograde movements of society. Political philosophy can make few steps without an occasional recourse to its aid, and none at all without a reference to its stores, on explaining the principles which regulate the population of states. Malthus, who may be in some degree considered as the father of that subject, from the maturity to which he has reared it, remarks, that 'we may promise ourselves a clearer insight into the internal structure of human society from those enquiries. But the science may be said yet to be in its infancy, and many of the objects, on which it would be desirable to have information, have been either omitted, or not stated with sufficient accuracy.'" vii.

Some of these inaccuracies have been corrected since the time when Malthus wrote, and our intelligent author modestly states that he should be amply rewarded if the present humble Essay should form a repository of the labours of his predecessors—"if it should become an early mile-stone on a road which is comparatively new, rugged, and uninviting to the traveller, which gradually discloses the most interesting prospects, and will, at length, largely recompense the patient adventurer."

The work is divided into sixteen chapters, and we shall endeavour to give a rapid sketch of these or most of them.

CHAP. I. This chapter embraces a comparison between the value of life in ancient and modern times. The author sets out with applying a piece of very flattering unction to the souls of modern Æsculapii.

"Medical statistics afford the most convincing proofs of the efficacy of medicine: it is one of the easiest arguments that can be employed to refute the vulgar notion (and one sometimes carelessly countenanced by medical men), that nature is alone sufficient for the cure of disease, and that art as frequently impedes as it accelerates her course. The powers of self-restoration are in no diseases more conspicuous than in fever. But if we form a statistical comparison of fever treated by art, with the results of fever consigned to the care of nature, we shall derive an indisputable conclusion in favour of our profession.* Hippocrates has left a frank and explicit statement of the history and fate of forty-two cases of acute disease, in which it does not seem that any therapeutical plan was adopted, if we except glysters and suppositories in a few, and blood-letting in one. Amongst these were thirty-seven cases of continued fever, without local affection. Of the thirty-seven, twenty-one died, above half of the whole. But if we examine the returns of the Fever Hospital of London, we find (in 1825) that the total mortality was less than one in seven; and half of these deaths occurred within seventy-two hours of the admission of the patients,—a circumstance which indicates that several entered at a period of disease when the hope of recovery was extinct. In the Dublin Fever Hospital we find a still lower mortality: the average from 1804 to 1812 was one in twelve: and in the clinical wards at Edinburgh, in 1818, the mortality of fever was also about one in twelve. Of five cases of local inflammations, which Hippocrates records, four were fatal; of all his forty-two patients, in short, twenty-five were lost: a termination which throws no shade over his skill, but only brings to light his love of truth. The mortality belonged to the age, and not to the physician; and we may reasonably infer, that under other practitioners of his time and country, it was even more severe. It is curious to observe, that of the five cases of local inflammation, the only one which survived was the solitary instance in which bleeding was employed,—a pleurisy. We perceive, that one out of two acute cases may recover by the almost unassisted efforts of nature, but that under the medical protection of our own age and country, six out of seven, or even eleven out of twelve, are likely to survive, according to the period of the disease at which they are placed under treatment." 5.

We shall not stop to inquire whether a comparison of the practice of Hippocrates with that of the fever-hospitals of London and Dublin be calculated to let in much light on the influence of modern medicine on the prolongation of human life. To form a just estimate, we must take into consideration the benefits arising from quackery, and also from the unavoidable mistakes of the best informed practitioners. If the true state of the case could be seen with these deductions, we should tremble to look at the picture!

Dr. H. instances the influence of various mechanical improvements on the air of certain districts, as the Island of Portsea, which was freed from agues by draining. Unfortunately the last two or three years have shewn a return of agues, not only in the best drained places, but in localities where ague had not been known in the memory of man before. The mortality at Portsmouth in 1800, was 1 in 28—in 1811 it was only 1 in 38—while at Plymouth, which is considered so remarkably healthy, it was 1 in 28.

We have no correct information respecting the longevity of the Greeks—and but scanty materials as to that of the Romans. In a small tract of Italy, there is an enumeration of 54 persons who had attained the age of

* Blane, Select Dissertations.

100—forty who were between 100 and 140—and two individuals who had got beyond 150 years! Dr. H. says this calculation is highly favourable to the longevity of the Romans, but it only relates to a "particular and rural district." True. But could any particular or rural district in England present such a spectacle?

Lucian has recorded that out of 1000 persons who died, 398 were above 60 years of age. This is considered a vague assertion, and is opposed by the testimony of Domitius Ulpianus.

"This earliest authority on the subject of longevity was a lawyer in the reign of Alexander Severus, of whom he became the secretary and principal minister. From the want of hospitals among the Romans, from the humble condition of their medical attendants, from their gross sensuality, inactive habits, abuse of the bath, and manner of dress, as well as from the unhealthy state of their situation (which even then appears to have been a source of alarm), we might have anticipated that longevity would not be common; and the authority of Ulpian corroborates the opinion. According to him, registers of population, puberty, age, sex, disease, and death, were kept with exactness by the censors, from the time of Servius Tullius to Justinian, and comprehend a period of ten consecutive centuries. But, unfortunately, these registers embrace the citizens of Rome alone, and not that large part of the population composed of slaves. The inferences to be drawn from them relate accordingly to select, or *picked* lives, and not to the mass of society. From observations formed on 1000 years, the expectation, or mean term of Roman life, has been fixed at thirty years. To make a just comparison of the value of life in Rome and in England, we must select subjects in England similarly circumstanced, of a condition relatively easy; and the result discloses an extension of life remarkably in our favour. Mr. Finlayson has ascertained, from very extensive observation, on the decrement of life prevailing among the nominees of the *tontines*, and other life-annuities granted by authority of Parliament, during the last forty years, that the expectation of life is above fifty years for persons thus situated, which affords our easy classes a superiority of twenty years above the Roman citizen. The expectation of life for the whole mass of Britain is at least one in forty-five, which affords to all our classes a superiority of fifteen years above even the easy classes of the Romans.

"The mean term of life among the easy classes of Paris is at present forty-two, which gives them an advantage of twelve years above the Romans." 7.

The expectation of life in Rome in the third century of the Christian æra was as follows:—From birth to 20, there was a probability of 30 years—from 20 to 25, 28 years—from 25 to 30, 25 years—from 30 to 35, 22 years—from 35 to 40, 20 years—from 40 to 45, 18 years—from 45 to 50, 13 years—from 50 to 55, 9 years—from 55 to 60, 7 years—from 60 to 65, 5 years. The computation did not extend beyond this.

It is certainly gratifying to compare the above with the results of modern researches on the probability of human life. At 20 years of age, Mr. Finlayson shews us a probability of 40 years—at 40, he allows 29 years—at 50, he promises 22 years—at 60, he admits 15 years!

"At Geneva, good mortuary tables have been preserved since 1560, and the results are in the highest degree curious and satisfactory. It appears that, at the time of the Reformation, half the children born did not reach six years of age; in the seventeenth century, the probability of life was about eleven and a half years; in the eighteenth century, it increased to above twenty-seven years. We arrive at the remarkable conclusion, that, in the space of about three hundred years, the probability of life to a citizen of Geneva at his birth, has become five times greater. The *mean life* was thus, in one century, eighteen years; in the next, it grew to twenty-three; in the middle of the next, it rose to thirty-two; and, finally, during the present century, from 1815 to 1826, it amounts to thirty-six years." 10.

Such a cheering prospect would almost induce the hope of immortality, if things went on thus improving. But, alas! the final period is still the same, although more reach the utmost goal than formerly.

CHAP. II. In the second chapter of the work, our indefatigable author adverts to the progressive changes and present state of mortality in Great Britain. We can only glance at a few of the particulars. In 1780, the annual mortality of England and Wales was 1 in 40. In 1790, it diminished to 1 in 45. In 1801, it continued to diminish, but not at the same rate—it became 1 in 47. This moderate rate of improvement is attributed to the scarcity which afflicted England in 1795 and 1800. In 1811, the ratio of death was 1 in 50—and finally, in 1821, the annual mortality sinks to 1 in 58 or 60. Thus it has decreased from 1 in 40 to 1 in 58 in the course of forty years.

There is great difference in the ratio of mortality in the different counties of Great Britain, varying from 1 in 47 to 1 in 72, Middlesex and Sussex being the two extremes. Pembrokeshire and Anglesey have only 1 death yearly in 83 individuals—the lowest rate of mortality that has been known in Europe. The mortality in each county is mainly influenced by the proportion of large towns which it includes.

"In Lincolnshire, the amount is only 1 in 62, although it is particularly the seat of ague; but this moderate share of mortality is probably due to the large proportion of dry and elevated districts to the fenny; if not to the circumstance which Dr. Wells has remarked, that phthisis pulmonalis is but little observed in places infested with the exhalations which produce intermittent fever." 17.

But the decline of mortality is even greater in our cities than in the rural districts. In London, the total deaths, in the year 1697, were about 21,000—whereas in 1797, the amount was only 17,000. This decrease in the absolute mortality is great, when we consider the immense augmentation of the metropolis in a century. It is remarkable that this healthy condition of London seems to have been chiefly the produce of the last 50 or 60 years. In the middle of the last century the annual mortality of the metropolis was 1 in 20—it is now 1 in 40. Thus the chances of existence have just doubled in the course of 70 years—a progress unparalleled in the history of any other age or country.

"One city alone, in Europe or in England, approaches to London in the value of life proportionately to its size; it is the second in England in number of inhabitants, the seat of manufactures—Manchester. The mortality of Manchester was, about the middle of last century, 1 in 25; in 1770, 1 in 28. Forty years after, in 1811, the annual deaths are diminished almost beyond belief, to 1 in 74; but the improvement does not stop even there, for, in 1821, they appear to become still fewer, although the population has been quadrupled during the 60 years through which the deaths have so diminished. It is due to the memory of Dr. Percival and Dr. Ferriar, that we ascribe a large share of this improvement of health to certain regulations of police, particularly with respect to ventilation, recommended and introduced by them into Manchester." 20.

### CHAP. III.—SUPERIOR SALUBRITY OF ENGLAND.

In France, the annual deaths in 1781 were 1 in 29 for the whole population—in 1802, they were 1 in 30—in 1823, 1 in 40. In Paris, about the middle of last century, the mortality was 1 in 25—at present it is about 1 in 32. It has been calculated that, in the 14th century, the mortality in Paris was 1 in 16 or 17. In Sweden, the range of mortality has decreased from 1 in 35 (1755 to 1775) to 1 in 48. Berlin has augmented in salubrity during the last 50 or 60 years, from 1 in 28 to 1 in 34.

"Since the late peace, the principal governments of Europe have paid much attention to statistics, and we possess very instructive returns from nearly all the countries, cities, and hospitals on the Continent. A comparison of these results enables us to submit a very interesting conclusion, and one which we are not aware to have been as yet generally received, namely, that the mortality of Great Britain, its cities, and its hospitals, is greatly inferior to that of any other country in Europe; and that it is incontestible that Great Britain is at present the most healthy country with which we are acquainted; and that it has been gradually tending to that point for the last 50 years. In the comparisons which we shall have occasion to make, in order to support this assertion, we shall carefully abstain from reproducing the tables of remote periods, which have been often previously discussed, and shall be confined to the most recent and genuine details. It is remarkable, that this superior value of life in Great Britain is not confined to any particular districts, or classes of individuals. To whatever point we turn our view, the advantage is still the same: the man of affluence, the pauper-patient of the hospital, the sailor and the soldier on active service, the prisoner of war, the inmate of a gaol, all enjoy a better tenure of existence from this country than from any other of which we have been able to consult the records. It has been long the fashion, both abroad and at home, to exhaust every variety of reproach on the climate of our country, and particularly on the atmosphere of London; and yet we shall find that the most favoured spots in Europe, the places which have long been selected as the resort of invalids, and the fountains of health, are far more fatal to life than even this great metropolis." 31.

The annual deaths, on the average, throughout the whole of England and Wales, is nearly 1 in 60. The country which approaches nearest to us in salubrity, is the Pays de Vaud, where the average mortality is 1 in 49. Sweden and Holland present the same standard nearly, 1 in 48. Next on the list is France, where 1 dies annually out of 40—a proportion precisely similar to that of London. The kingdoms of Prussia and Naples follow—

ranging from 1 in 33 to 35. The annual mortality at Montpelier is greater than in London, which equals in salubrity the department of the HERAULT, "the southern, the fertile, and the long-supposed most salubrious district of France, of which Montpelier is the capital."

"Finke, a German writer who wrote on medical geography in 1792, speaks with surprise and reprobation of the custom which then prevailed in England of sending invalids to the south of France; and declares that the cutting winds of those quarters annually destroyed many of those wanderers in quest of a milder sky.

"The annual mortality of Nice, though a small town, and enjoying a factitious reputation of salubrity, is 1 in 31; of Naples, is 1 in 28. Leghorn is more fortunate, and sinks to 1 in 35. We instance those places as being the frequent resort of invalids; but how astonishing is the superiority of England, when we compare with these even our great manufacturing towns, such as Manchester, 1 in 74; such as even Birmingham, 1 in 43; or even this overgrown metropolis, where the deaths are only 1 in 40. But if we take indiscriminately the other great cities of Europe, their inferiority in respect to the value of life is equally pointed; in Paris, for instance, the annual deaths are about 1 in 32, in Lyons and Strasburg the same, in Barcelona the same: Berlin approaches a little nearer to London, it reckons 1 in 34. Madrid loses 1 in 29. Rome, Amsterdam, and Vienna, are last in the scale of life; in Rome the deaths are annually 1 in 25, at Amsterdam they are so numerous as 1 in 24, and at Vienna 1 in $22\frac{1}{2}$: we perceive that the inhabitant of London has almost a twofold advantage in this respect." 33.

We may remark upon the above passage that, although the mortality may be greater in Rome, or Naples, or Nice, than in London, it does not follow that particular complaints, as incipient pulmonary affections, may not be benefited by a *temporary* residence during a *particular season* of the year in the above localities. A man may die of heat and malaria at Rome in the month of August—but his life may be preserved by a residence there during December, January, and February, in consequence of the mildness of the air, compared with Hampstead or Highgate. It is hardly fair, therefore, to laugh at the invalid who migrates during the Winter or Spring to a milder climate than that of England, though the latter, taking the whole year round, may be more salubrious than the climates of the most favoured spots on the Continent.

### CHAP. IV.—MEDICAL STATISTICS OF COUNTRIES.

FRANCE. It is calculated that about half of the children born live to 20 years, and a third to 45 years. The lowest annual mortality is at the age of 10, when it is only 1 in 130. At the age of 40 it is 1 in 53. The probability of life in France, at the age of 40, is 23 years. The mortality increases amongst the poor and diminishes among the affluent. In the wealthy departments of France, life is protracted 12 years beyond its course in those which are poor. Thus, in the departments of the Calvados of L'Orne and La Sarthe, one individual dies annually in 50; while, in the twelfth Arrondissement of Paris, the annual deaths are 1 in 24!! Such is the influence of condition, or the absence of fatigue and privation on the frame, that, during the years 1816 to 1823, the mean height of young men fit for military service has been found to be, in Paris, 5 feet, 2 inches, and $1\frac{1}{2}$ lines—but only 5 feet, 1 inch, and $9\frac{1}{2}$ lines for the suburbs of Sceaux and St. Denis. The same fact has been ascertained in Lyons and its vicinity.

PRUSSIA. One birth in 13 is illegitimate in Prussia. The average mortality is about 1 in 35.

AUSTRIA. A sixth of the whole population is illegitimate in the province of Styria. The mortality is 1 in 38. In *Russia*, we find the mortality 1 in 41. In America (United States), it is calculated that, on an average, 1 in 40 die annually—the rate of France. In South America, Humboldt calculated the average mortality at 1 in 30, which is a greater proportion than now occurs in any country of Europe.

### CHAP. V.—MEDICAL STATISTICS OF CITIES.

"It is well known, that in any given country the deaths of a city are more numerous than those of the rural districts. This difference is principally felt in the first five years of life, when many more die in London than in the country. From 5 years of age to 20 the deaths in London are fewer. Between 20 and 50 many more die in London, on account of the large annual influx from the country. In all cities a large portion of disease and death is to be assigned to the constant importation from the country of individuals who have attained to maturity; but having been previously habituated to frequent exercise in a pure atmosphere, and to a simple regular diet, are gradually sacrificed to confined air, sedentary habits, or a capricious and over-stimulating food. These causes are not equally fatal to those who have passed their early years within the walls of a city; and after the age of 50 the proportion of deaths in London is smaller than in the country. Jenner, and very recently Dr. Baron, have made some curious experiments on animals, which indicate that a loss of their open range and natural nourishment has with them, also, a tendency to disorganize and to destroy. Dr. Baron placed a family of young rabbits in a confined situation, and fed them with coarse green food, such as cabbage and grass. They were perfectly healthy when put up: in about a month one of them died: the primary stop of disorganization was evinced in a number of transparent vesicles studded over the external surface of its liver.

"In another, which died nine days after, the disease had advanced to the formation of tubercles on the liver. The liver of a third, which died four days later still, had nearly lost its true structure, so universally was it pervaded with tubercles. Two days subsequently a fourth died: a considerable number of hydatids were attached to the lower surface of the liver. At this time Dr. Baron removed three young rabbits from the place where their companions had died to another situation, dry and clean, and to their proper and accustomed food. The lives of these remaining three were obviously saved by this change. He obtained similar results from experiments of the same nature performed on other animals." 55.

In GLASGOW, the average annual mortality is about 1 in 44 persons. In

Paris, the poor and the rich occupy the two extremities of the scale. The mortality in the one is nearly double that in the other. The average is 1 in 32.

"The number of violent deaths in 1823 was 690, of which 390 were cases of suicide.

"Reviewing, on one side, the great political, moral, and physical events, which have occurred at *Paris* during a succession of years, and on the other the progress of its population, Villermé has ascertained, that whenever the people have suffered from *any* cause, the deaths have correspondingly increased, the births have decreased, and the mean duration of life has been shortened. In periods of prosperity he has found results directly opposite to these. The mean duration of life in Paris is 32 years and some months.

"It was formerly estimated, that one third of the inhabitants of Paris died in the hospitals; but Dupin has lately calculated that half the deaths in Paris take place in the hospitals and other asylums of charity. Not a fourth part of the inhabitants are buried at private cost." 59.

In GENEVA, the average mortality for the four years ending in 1823, was 1 in 43—which is a much greater mortality, by the way, than in some of our largest manufacturing towns, as Glasgow, Manchester, and Birmingham.

PETERSBURG. It is curious that the burials exceed the births in the Russian capital by 134 to 100. The Russians attempt to explain this by the annual influx of persons from the provinces. But this influx is not peculiar to St. Petersburg. The last-mentioned city and Stockholm are the only known metropolitan cities which present the preponderance of death over production. The annual mortality of the Russian capital is one in 37.

"BERLIN. From 1747 to 1755, the annual mortality of Berlin was 1 in 28. Between 1796 and 1799, it improved to 1 in 29$\frac{1}{11}$. Here the beneficial change was retarded by the ravages, the losses, the disappointments of war, and from 1802 to 1806 it had retrograded to 1 in 27; but from 1816 to 1822, a period of exultation and tranquillity to the Prussians, the value of life took a remarkable leap, and the annual deaths fell to less than 1 in 34." 61.

VIENNA. In the middle of the last century, the mortality of Vienna was 1 in 20; and it has not improved in proportion as other cities of Europe. According to the most recent calculations, it is, even now, as 1 in 22½. Among 10,530 deaths, scarcely 38 persons are found who have attained the age of 90.

"The excessive spirit of regulation, the dread of novelty, the restrictions imposed on the medical profession, and political causes which need not to be enumerated, appear to have retarded the natural progress of this city. The overweening *paternity* of the government interferes with the trivial concerns of the citizens, in the same manner in which an arbitrary and untaught father sometimes restrains the useful impulses of his children, while he permits an easy vent to their baser propensities." 63.

PRAGUE, the capital of Bohemia, has only one-third the population of Vienna, and is much healthier. The superior longevity of the JEWS is strongly marked in this city. One death is annually observed among 26 of the Israelites, while it is 1 in 22½ among the Christians. Instances of considerable longevity, especially among the women, are not rare. Contrary to the usual observation, longevity is confined to poverty, and married life.

"According to an average of several years no nobleman, no wealthy person, no bachelor, and no unmarried woman have *passed* the age of 95. This is an interesting fact, but it is an *extreme* and an insulated one, and does not militate against the general *conservative* tendency of prosperity which a variety of evidence seems to establish." 64.

PALERMO. Mortality here is 1 in 31. January, October, and November, are the most fatal months—April May, and June the most healthy.

LEGHORN. The average annual mortality here is 1 in 35—among the Protestants and Jews it is only 1 in 48, which is attributed to their greater affluence.

ROME. From a recently-discovered fragment of Cicero (de Republica,) an intimation is conveyed that the neighbourhood of Rome has been always unhealthy. Speaking of the choice of situation made by Romulus, he observes—"locum delegit in regione pestilente salubrem." The population appears to have been gradually decreasing till the last peace, which has gently revived it. In 1800 there were 150,000 souls—in 1810, only 123,000—within a few years it has gained 10,000. The annual mortality is about 1 in 25.

"There can be little doubt that the force of the aguish disposition of Rome might be considerably weakened by steady and well-directed efforts supported by a proportionate capital; but it is to be feared that such a combination of circumstances will not readily meet at Rome. In 1816, 17 out of the 22 French students were attacked with intermittent fevers. The Villa Medici, in which they reside, was formerly healthy; but water brought at a great expense to embellish the garden had been suffered to stagnate there." 67.

NAPLES. The annual mortality here is 1 in 28—a fact that one would not have expected in such a delightful situation, compared with pestilential Rome, where the mortality is less. The population of Naples is nearly three times that of the ancient mistress of the world!!

BRUSSELS. It appears from our author that, at Brussels, "the month of May is the most favourable for conception." The average mortality is very great, being 1 in 26.

AMSTERDAM. The population of this once great city is decreased, in consequence of declining commerce and political changes. And it is not a little curious, as well as melancholy, to observe that its mortality has increased with the progress of decay. In 1777, the ratio of mortality was 1 in 27—a period when Amsterdam was one of the healthiest, as well as one of the most flourishing cities of Europe. The deaths have now increased to 1 in 24—and Amsterdam is one of the least healthy, as well as least prosperous, sea-ports of Europe. A decree has been issued, that after the 1st of January, 1829, no burials shall be permitted in towns or in churches throughout North Holland.

STOCKHOLM. "Drunkenness appears here, as at Berlin, to produce a large share of the mortality. In a recent year this city exhibited a singular instance of an excess of 1439 more deaths than births; a symptom which it is painful to observe in a brave and industrious people. This disproportion existed particularly amongst the garrison, and is ascribed to immoderate use of brandy. Our authority affirms that this vice destroys the happiness and prosperity of Sweden more effectually than any war has ever accomplished." 70.

### CHAP. XI.—SUICIDE IN DIFFERENT PERIODS AND COUNTRIES.

Being obliged to pass over some chapters on the statistics of hospitals, we shall dwell, for a moment, on the melancholy subject of suicide.

Dr. Burrows has the merit of having first vindicated our country from the conjectural report of a peculiar tendency to self-destruction. The bills of mortality, on which Dr. Burrows founded his calculations, have been objected to by some foreigners, on account of their inaccuracies:—But a document has been lately published which enables us to satisfy the most fastidious sceptic on that point.

"It is well known that a coroner and jury are summoned to investigate every suicide which occurs in Westminster. Mr. Higgs, the coroner of the city of Westminster, made, in 1825, a report of the suicides committed in Westminster during the 13 years previous. In order to furnish easy means of comparison, we must premise that the population of Westminster, according to the census of 1821, was 182,444. We may add, for the use of strangers, that it is the centre of dissipation for the whole empire. During the 13 years from 1812 to 1824, the *total* number of suicides was only 290, a number which, *if trebled*, would be inferior in proportion to the returns made by the great cities of France and Germany.

"The number of males in this table is 207, of females only 83, which is a proportion of 5 to 2. The *Novembers* of these 13 years produce only 22, while the rate of the *Junes* is 34. In the years 1812, 1815, 1820, and 1824, November did not afford one suicide. The least prolific months were May and September, next August and October, and then November. During the latter eight years, a reduction occurred on the average of nearly six per annum. The annual average is a little more than 22 during the whole term. From motives of humanity, the juries gave a verdict of *insanity* in all but five instances. In 1825, a year marked by commercial distresses, the total number was 24: of these, two women had been seduced and abandoned, and one man cut his throat through jealousy, eight poisoned themselves, and eight were found hanging.

The annual number returned by the bills of mortality for London usually ranges between 30 and 50. After making every allowance, we may estimate the number of suicides annually accomplished in London and Westminster at about 100. In England and France a majority of the victims appear to be *unmarried*. In France, the proportion of married men to single amongst suicides has been rated as only 2 to 3.*

"Other countries certainly present a darker picture. We are not surprised at finding the number of 1300 recorded for Versailles in 1793, a year of political storm, and of dreadful anticipation to its inhabitants. In 1806, Falret asserts that the suicides of Rouen amounted to 60 during the months of June and July alone. Professor Grohmann notes a remarkable increase at Hamburgh:—in 1816

* "Dict. Sciences Medic. art. Celibat."

the number was only 2, in 1820 it rose to 10, and 1822 produced so many as 59. In the small district of Frankfort on the Main the number in 1823 was 100.

"In 1806, there were 300 at Copenhagen :—of late years the annual average has been 100 in 100,000 inhabitants. In Berlin, according to Casper, the proportion is 34 annually in every 100,000 inhabitants, and in Paris 49. The increase in Prussia, and particularly in Berlin, is extraordinary. In the 17 years following 1758, the proportion at Berlin was 1 suicide in 1800 deaths. But in the 10 years following 1787 the proportion is seen to double itself, becoming 1 in 900 deaths. In the 10 years following 1798, it is trebled; and in the 10 years ending in 1822 it arose to the formidable height of 1 in every 100 deaths. These numbers, large as is their amount, do not include many who are found drowned in the river, and whose fate is dubious. In 1817, the proportion for the whole Prussian nation was 1 in every 400 deaths. We must remark on the comparative frequency of this crime amongst *boys* in France and Germany. We should not venture to state the curious fact of the existence of a *suicide club* in Prussia, except on the authority of Dr. Casper, an eminent statistical writer resident in Berlin, a city where every work is submitted to the *censure*. This club consisted of six persons, who avowed openly their intention of destroying themselves, and endeavoured to gain proselytes. Their absurdity excited more laughter than belief, but three instances occurred of conformity to principle, and at length all the six evinced their sincerity; the last shot himself in 1817." 165.

In Berlin, suicide appears to be more frequent among weavers and soldiers than in other classes of society, It is more common among the females of Paris than among those of Berlin, in a two-fold average—a fact that might be anticipated from the more retired and unambitious path of the German woman. During six recent years, 18 cases of suicide happened at Berlin, under the age of puberty—and 11 men above 70. So far more numerous are the civic than the rural cases, in Prussia, that while the proportion in towns is 14 in every 100,000 inhabitants, the country exhibits only 4 in the same number. This crime appears to find very few victims in Spain. In the whole of that country only 16 instances are officially reported to have occurred in the year 1826. In all Sweden there were only 153 suicides in the year 1823. In four years (1823-4-5-6) 4087 suicides took place in Russia!

"A *suicide club* is *said* to have existed lately at Paris, but the members were not likely to become numerous: they were 12, and the leading regulation directed, that one member should be annually selected to put an end to himself.

"Among Roman Catholics the disposition to suicide appears far less prevalent than in Protestant communities. It would be easy to dilate on the sources of this disproportion. Blumerbach made the observation in respect to Switzerland, and Casper has established it relatively to Germany. It is very rare among the *Jews* of Germany, partly from the dread of ridicule which disinclines them towards taverns, and partly from the beneficence of the wealthy members towards the indigent of their own race.

"Climate, then, cannot be considered as a cause, and no one will hereafter ascribe it to changes of weather." 171.

Passing over several chapters, we offer the following extract from the chapter on Climatology.

"When we speak of a healthy climate, it is gratifying to reflect that in most instances it is man himself who has in a great measure created these climates of health. Twenty centuries ago, England, France, and Germany, resembled Canada, and Chinese Tartary, countries situated like Europe, at a mean distance between the equator and the pole. Macchiavelli, in his early age, seems to have anticipated this truth: he remarks, in his quaint language, 'Unhealthy countries become wholesome by the multitude of men who inhabit them; who at the same time are occupied in cultivating the earth, and who make the earth sane: the fires which they kindle purify the air: these advantages nature herself does not produce.'

"It is only by constant efforts of industry that the salubrity of any spot is maintained: when these are relaxed, or when prosperity and civilisation decline, the seeds of disease are immediately deposited in the earth. The aguish disposition has been observed to increase at Rome in the same proportion that its population has diminished. On the other hand, it is well known that the climate of the United States has been remarkably improved by draining, cutting down trees, and the operations of agriculture; and that spots which were impracticable, or fatal to the early settlers, at present afford a comfortable residence. The improvement that is continually taking place in the climate of America proves that the power of man extends to features of nature, which from the magnitude and variety of their causes seemed entirely beyond his control. At Guiana, in South America, within five degrees of the line, the inhabitants, living amidst immense forests, were a century ago obliged to alleviate the severity of the cold by evening fires. But by clearing the surface of the country even the duration of the rainy season has been shortened, and the warmth is so increased that a fire would now be deemed an annoyance. It thunders continually in the woods, but rarely in the cultivated parts.

"It appears certain that the climate of Europe has undergone a great change. If we compare its actual state with the accounts of ancient writers, a remarkable discrepancy is observed, which can only be explained by the influence of industry on the improvement of the soil; and there is reason to believe that America will partake of the same happy amelioration when an equal length of gradual toil has been bestowed upon her. We are told by Cæsar, that the vine could not be cultivated in Gaul on account of its winter-cold. The rein-deer, now found only in the zone of Lapland, was then an inhabitant of the Pyrenees. The Tiber was frequently frozen over, and the ground about Rome covered with snow for several weeks together, which almost never happens in our times.

"Even on nations exposed to the same scorching sun the influence of *diet* seems to be more powerful in forming the constitution and the character than mere climate, as is evinced in the wide diversity existing between the Hindoo and the Malay. The *nature of the soil* is the earliest element which operates in creating a national character; but religion and government produce a second, a more essential, a moral climate, which ultimately determines not merely the health of citizens, but the existence of a state." 205.

The chapter on the influence of condition, profession, modes of life, &c. on longevity, contains much interesting information which we are obliged to pass unnoticed, in consequence of our narrowing limits. It was long a prevalent opinion that poverty was conducive to long life; but the contrary is evidently the fact. Of an equal number of infants taken from among the poor and the easy classes, it will be found (at least it has been found in France, where the question has been the most agitated) that the proportion of deaths among the former is double:—and that wherever is the greatest portion of misery, there will also be found the largest share of mortality.

"The conservative tendency of an easy condition is strongly marked by the very inferior degree of mortality and of disease which occurs among persons insured at the various life-offices. The Equitable Office had always employed the corrected Northampton tables of the probabilities of life. But Mr. Morgan, the actuary, found in 1810 that the actual deaths which had occurred among 83,000 persons insured during 30 years was in the proportion of only 2 to 3 of what had been anticipated by the tables. And among these *selected* lives the mortality of the women is still less than that of the men; because in the middle classes they enjoy a remarkable exemption from fatigue and difficulty. To illustrate the low rate of mortality among such picked lives, or among persons in the enjoyment of competence, it may be mentioned, that the annual average of deaths amongst the persons insured at the Equitable from 1800 to 1820 was only about 1 in 81½. Of 1000 members of the University Club, only 35 died in 3 years, which is a still lower rate, about 1 in 90 annually. Of 10,000 pupils who passed in different years through Pestallozzi's institution in Switzerland, it is even asserted that not one died during his residence there. These were youths chiefly, but of all countries, constitutions, and ages; generally, it is to be observed, of easy circumstances. Pestalozzi, also, paid particular attention to their bodily exercises." 209.

Dr. Hawkins concludes the work with a chapter on the application of medical statistics to the illustration of the principle of population.

After enumerating the many varieties in the distribution of mortality, Dr. H. shortly inquires into the causes which diminish it, and especially in this country.

"Among the general causes, the increase of commercial and agricultural industry has multiplied the comforts of the lower classes, and has enabled them to procure a more spacious dwelling, more frequent changes of clothing, and more abundant and more wholesome food; insomuch, that the average mortality and health of every nation are mainly determined by the degree in which its government has encouraged these pursuits, or has checked their free course. So intimate a connection subsists between political changes and the public health, that wherever feudal distinctions have been abolished, wherever the artisan or the peasant have been released from arbitrary enactments, there also the life of the lower classes has acquired a new vigour; and it is certain, that even bodily strength and the power of enduring hardships are divided among the nations of the earth in a proportion relative to their prosperity and civilization.

"We may easily conceive the different constitution of body and of mind which is likely to grow upon the unemployed inhabitant of a decayed city, who gloomily wanders without an object through silent streets whose pavement is choked with grass; and upon the active citizen who feels himself a constituent member of a flourishing community, and who is attracted on all sides by invitations to the exercise of his faculties.

"It is indisputable, that the average proportion of deaths in England and her cities is less than that of any other country of Europe. And it may be added, that the powers of body and of mind are preserved to a late period in higher perfection here than in other countries: nowhere are the advances of age so slowly perceived, and nowhere so little manifested on the exterior. An analogous condition of health and vigour may be also observed in our animals and in our vegetation; and if it should be replied, that this excellence is owing to the care bestowed on their culture, the answer applies equally to the human being, on whom more attention is here bestowed, and who is really an object of greater value here than elsewhere." 233.

We cannot part with our talented and erudite author, without expressing our admiration of his industry, his good sense, and his amiable feeling, as evinced through every chapter of the work under review. We shall be much deceived, as well as disappointed, if other, and still more interesting productions, do not ultimately issue from the same source. *Perge pede quo cœpisti.*

